# Relationship between tracheal intubation and the drugs used by patients with drug overdose due to self-harm

**DOI:** 10.1186/s40780-021-00234-7

**Published:** 2022-01-03

**Authors:** Kazuki Nagashima, Hiroyuki Hosono, Machiko Watanabe

**Affiliations:** Laboratory of Clinical Pharmaceutics, Faculty of Pharma-Science, Teikyo University, 2-11-1 Kaga, Itabashi-ku, Tokyo, 173-8605 Japan

**Keywords:** Overdose, Tracheal intubation, GCS, JCS, Antipsychotic, Anticonvulsant, Blood concentration

## Abstract

**Background:**

Tracheal intubation may be performed in patients with drug overdose due to self-harm; however, the details of the causative drug are unknown. The purpose of this study was to clarify the relationship between drugs or its blood levels of patients with drug overdose and the need for tracheal intubation based on the actual measurement results.

**Methods:**

From October 2018 to March 2020, 132 patients with drug overdose due to self-harm who were transported to the emergency department (ED) were studied. Patient drugs were measured using gas chromatography–mass spectrometry (GC-MS) and were analyzed on the basis of the GC/MS Forensic Toxicological Database. Logistic analysis was performed by combining patient information and GC-MS information.

**Results:**

The Glasgow Coma Scale (GCS) and Japan Coma Scale (JCS) efficiently predicted tracheal intubation in patients with drug overdose during transport triage; GCS (cut-off value: 12, area under the curve (AUC): 0.81, 95% confidence interval (CI): 0.71–0.88, sensitivity: 0.85, specificity: 0.71, *P* < 0.05) and JCS (cut-off value: 3, AUC: 0.74, 95% CI: 0.60–0.84, sensitivity: 0.60, specificity: 0.84, *P* < 0.05). The drugs detected in all patients with drug overdose in order were benzodiazepine receptor agonists (BZs; 43.9%), anticonvulsants (38.6%), antipsychotics (25.0%), and antidepressants (9.8%). In univariate logistic analysis, antipsychotics (odds ratio (OR) 2.46, 95% CI 1.19–5.20, *P* < 0.05), anticonvulsants (OR 2.71, 95% CI 1.26–5.98, *P* < 0.05), and anticonvulsants above alert blood levels (OR 27.8, 95% CI 2.92–264.1, *P* < 0.05) were significantly associated with tracheal intubation in patients with drug overdose, but not BZs and antidepressants. Also, in multivariate logistic analysis, antipsychotics (OR 2.27, 95% CI 1.07–4.83, *P* < 0.05), anticonvulsants (OR 2.50, 95% CI 1.14–5.64, *P* < 0.05) and in multivariate logistic analysis of blood levels, anticonvulsants above the alert blood levels (OR 24.9, 95% CI 2.56–241.6, *P* < 0.05) were significantly associated with tracheal intubation in patients with drug overdose respectively.

**Conclusions:**

Logistic analysis revealed that the use of anticonvulsants and antipsychotics were significantly associated with an increased OR in the tracheal intubation of patients with drug overdose due to self-harm.

## Background

Tracheal intubation may be performed in patients with drug overdose due to respiratory failure and central nervous system depression caused by the action of drugs used by these patients [1, 2]. In addition, tracheal intubation is performed for patients with drug overdose due to various factors, such as cardiovascular toxicity and multiple organ failure [3, 4]. Tracheal intubation is important in securing the airway for breathing, but the criteria for tracheal intubation in patients with drug overdose are unclear [1]. There are few reports on drugs requiring tracheal intubation due to overdose. Information on drugs that increase the risk of tracheal intubation in patients with drug overdose may lead to rapidly saving the lives of these patients.

The Glasgow Coma Scale (GCS), which is used for consciousness judgment in the cranial nerves (traumatic brain injury), is used to determine the patient’s status [5, 6]. The GCS is determined by the total E (eye-opening), V (verbal), and M (motor) responses, and is used worldwide [7].

The Japan Coma Scale (JCS) was reported by Dr. Tomio Ohta in 1976 in Japanese as an index for determining the severity of impaired consciousness [8–10]. Since JCS uses only eye reactivity as an index to indicate the level of consciousness, it is easier and faster to determine the patient’s level of consciousness compared with GCS. The JCS is scored as 0 (Clear), 1 (Eye-opening spontaneously), 2 (Eye-opening to verbal or pain stimuli), and 3 (No eye-opening to any stimuli) [8, 9]. Also, in a cohort study, JCS is sufficient to predict stroke outcomes [10] and is reported to predict in-hospital mortality upon arrival at the hospital after trauma [11, 12]. However, there are few reports of how drug overdose is associated with GCS or JCS.

A study examined the number of reports from a database that registered self-reported drugs in patients with drug overdose who had tracheal intubation [13, 14]. However, there are no reports that statistically examined the relevance of tracheal intubation using measured values of patient samples. In addition, there are no reports of how gas chromatography–mass spectrometry (GC-MS)-identified drugs in patient samples is associated with the risk of tracheal intubation in patients with drug overdose. The purpose of this study was to measure the drugs or its blood levels of patients with drug overdose, identify the drugs that require tracheal intubation on the basis of logistic analysis, and evaluate the odds ratio (OR). The results obtained may be beneficial for transport triage in the emergency department (ED).

## Methods

### Patients and study design

From October 2018 to March 2020, 132 patients with drug overdose who were transported to Teikyo University Hospital were studied. The primary endpoint of this retrospective study was tracheal intubation. Patients with drug overdose were defined as patients who were diagnosed with drug overdose by doctors. Blood that were randomly left over at the hospital laboratory and anonymized were used as samples. Blood samples collected immediately after transport were used for the measurement. Psychotropic drugs (benzodiazepine receptor agonists (BZs), antipsychotics, antidepressants, and anticonvulsants) are among the leading causes of patients with drug overdose needing emergency transport [15] and were analyzed in this study. Drugs identified using GC-MS were evaluated. Other patient information was collected from electronic medical records. Information, such as temperature and pulse rate, used in this study was the value at the time of arrival at the hospital. To match the conditions of acquisition timing, there are some missing values (pulse rate, respiratory rate, PO_2_, time from patient discovery to sample collection and time from patient discovery to transportation). Table 1 specifies the number of defects.

**Table 1:** Characteristics of patients and GC-MS detected drugs

	Total	with tracheal intubation	without tracheal intubation	*P* value
n	132	20	112	
Sex (Male/Female)	27/105	6/14	21/91	
Age (average ± S.D.)	35.3 ± 14.9	42.3 ± 15.1	34.0 ± 14.5	0.01
**GC-MS detected**				
Antidepressants (n [%])	13 [9.8]	3 [15.0]	10 [8.9]	0.40
Antipsychotics (n [%])	33 [25.0]	11 [55.0]	22 [19.6]	0.0008
Anticonvulsants (n [%])	51 [38.6]	12 [60.0]	39 [34.8]	0.03
BZs (n [%])	58 [43.9]	8 [40.0]	50 [44.6]	0.70
**GC-MS detected (over the alert levels in blood)**				
Antidepressants (n [%])	4 [3.0]	0 [0]	4 [3.6]	0.39
Antipsychotics (n [%])	5 [3.8]	2 [10.0]	3 [2.7]	0.11
Anticonvulsants (n [%])	5 [3.8]	4 [20.0]	1 [0.9]	< 0.0001
BZs (n [%])	32 [24.2]	4 [20.0]	28 [25.0]	0.63
**GCS**				
E (average ± S.D.)	2.9 ± 1.2	1.9 ± 1.2	3.1 ± 1.1	< 0.0001
V (average ± S.D.)	3.6 ± 1.6	2.3 ± 1.3	3.9 ± 1.5	< 0.0001
M (average ± S.D.)	5.3 ± 1.3	4.4 ± 1.8	5.5 ± 1.1	< 0.0001
Total (average ± S.D.)	11.9 ± 3.7	8.5 ± 3.6	12.5 ± 3.3	< 0.0001
**JCS**				
0 (n [%])	3 [2.3]	0 [0]	3 [2.7]	0.46
1 (n [%])	58 [43.9]	4 [20]	54 [48.2]	0.02
2 (n [%])	41 [31.1]	4 [20]	37 [33.0]	0.25
3 (n [%])	30 [22.7]	12 [60]	18 [16.1]	< 0.0001
Body temperature (°C)	36.4 ± 0.87	36.6 ± 1.7	36.3 ± 0.6	0.50
Pulse rate (beats/min)	91 ± 26.5 *	106.4 ± 26.9	88.2 ± 25.4 *	(0.004)
Respiratory rate (breaths/min)	20.1 ± 8.9 **	21.5 ± 8.3 *	19.9 ± 9.0 ***	(0.43)
PO_2_ (mmHg)	155.6 ± 137.4 **	238.6 ± 166.2	140.1 ± 125.4 **	(0.58)
Time from patient discovery to sample collection (average ± S.D. [minutes])	62.1 ± 66.9 *	89.3 ± 104.9	57.1 ± 56.1 *	(0.53)
Time from patient discovery to transportation (average ± S.D. [minutes])	46.7 ± 42.4 *	42.6 ± 23.4	47.5 ± 44.9 *	(0.82)

*P* values were obtained from a chi-square test for categorical variables and Mann–Whitney U test for continuous variables, comparing with and without tracheal intubation groups

GC-MS detected and GC-MS detected (over the alert levels in blood) indicate total number

* 1 case unknown

** 5 cases unknown

*** 4 cases unknown

### GC-MS

GCMS-QP2020 (Shimadzu, Japan) was used for GC-MS. The column was Rxi-5SilMS 30 m × 0.25 mm (Restek Corporation, U.S.A.), and the upper limit of the column temperature was set to 320 °C. The carrier gas was helium, the MS ion source temperature was 200 °C, and the ionization method was electron ionization. The injection volume was 1 μL.

The GC/MS Forensic Toxicological Database Ver. 1.1 (Shimadzu) was used for data analysis [16]. An example of using this measurement system was previously reported [17]. In more detail, in the GC/MS Forensic Toxicological Database Ver.1.1, the retention time of GC and the fragment information of MS for drugs are registered in advance. The database can simultaneously measure the blood concentrations of 90 drugs semi-quantitatively. Psychiatric drugs and drugs of abuse are registered and are known to cause an overdose. The semi-quantitative analysis method looked for instances where multiple substances were simultaneously detected or when standard substances were rare, and many reports had been made [18–22]. The slope of the calibration curve is required for semi-quantification by this database. When measuring concentrations other than those registered, it is possible to adapt to a simultaneous quantitative system by creating a calibration curve y = ax + b for the target drug by the internal standard method and incorporating “a” (the slope) in the database. More than 500 kinds of drugs are incorporated in the database. Furthermore, the NIST14 library is also incorporated, making it possible to apply any toxicant and simultaneously perform qualitative analysis.

Standards were analyzed on the basis of four or five points (1–30 ppm). Measurement was performed using an internal standard method. Custom Internal Standard (Shimadzu GLC; 4-Chlorotoluene-d4, Acenaphthene-d10, 1, 4-Dichlorobenzene-d4, Fluoranthene-d10, Naphthalene-d8, Chrysene-d12, Phenanthrene-d10, and Perylene-d12) was used as an internal standard substance. The following drugs were used to add quantitative information to the database: Olanzapine, nortriptyline, paroxetine, clonazepam, sertraline, zopiclone, levetiracetam, and lamotrigine. Olanzapine, nortriptyline, paroxetine, levetiracetam, and lamotrigine were purchased from Tokyo Chemical Industry CO., LTD (Japan). Clonazepam and sertraline were purchased from Fujifilm Wako Pure Chemical Corporation (Japan). Zopiclone was purchased from Sigma-Aldrich (U.S.A.). The information of “a” (the slope) obtained were as follows: olanzapine (0.316), nortriptyline (0.571), sertraline (0.11), paroxetine (0.178), zopiclone (0.16), clonazepam (5.50), levetiracetam (0.897), and lamotrigine (0.127). The calibration curves were R^2^ > 0.99. The data were incorporated into the GC/MS Forensic Toxicological Database. Other drugs were semi-quantified using the preset values in the database.

### Pretreatment of patient blood samples and recovery rate of drugs

Patient samples were pretreated for purification before the measurement was performed. QuEChERS (Restek Corporation) and Q-sep dSPE (Restek Corporation) were used for pretreatment. The operation followed the product protocol. Nitrogen was blown to solidify the components. The samples were reconstituted with 50-μL ethyl acetate.

The Custom Internal Standard was set to 1 ppm to make measurement solutions and measured by GC-MS. The loss of pretreatment (QuEChERS-dSPE) was corrected by using the recovery rate of drugs [23].

### The alert levels in the blood

The alert levels of drugs in the blood were determined according to the literature [24–26]. The alert levels in the blood represent drug concentrations exceeding a certain level, where a significantly increased risk of concentration-related adverse drug reactions can be expected.

### Statistical analysis

The logistic regression analysis, receiver operating characteristics (ROC) curve, chi-square test, and Mann–Whitney U test were performed using JMP Pro. 15 (SAS Institute Inc., NC, U.S.A.). The significance level was set at less than 0.05.

## Results

### Patients and GC-MS-detected drugs

A comparison of the characteristics of each patient groups. Table 1 includes sex, age, GC-MS-detected drugs, GCS, JCS, body temperature, pulse rate, respiratory rate, PO_2_, time from patient discovery to sample collection and time from patient discovery to transportation. In total, 20 of 132 patients (15.2%) were of tracheal intubation. In the group with tracheal intubation, blood sampling may have been delayed due to various treatments by tracheal intubation. In patients with tracheal intubation, the average age was about 8 years higher than without tracheal intubation group. The drugs detected in all patients with drug overdose in order were BZs (43.9%), anticonvulsants (38.6%), antipsychotics (25.0%), and antidepressants (9.8%). The GC-MS-detected rate of BZs was almost the same with and without tracheal intubation; however, anticonvulsants, antipsychotics, and antidepressants were higher in patients with tracheal intubation.

Although there are some missing values, in patients with tracheal intubation,” time from patient discovery to sample collection” was about 30 min longer on average than in patients without tracheal intubation. The average pulse rate (beats/min) and respiratory rate (breaths/min) were high in patients with tracheal intubation compared with those without tracheal intubation. In addition, average PO_2_ (mmHg) was higher than the reference value in the group of patients with drug overdose and was remarkable in those with tracheal intubation.

### GCS and JCS at transport efficiently predicted tracheal intubation in patients with drug overdose

**Fig. 1:**
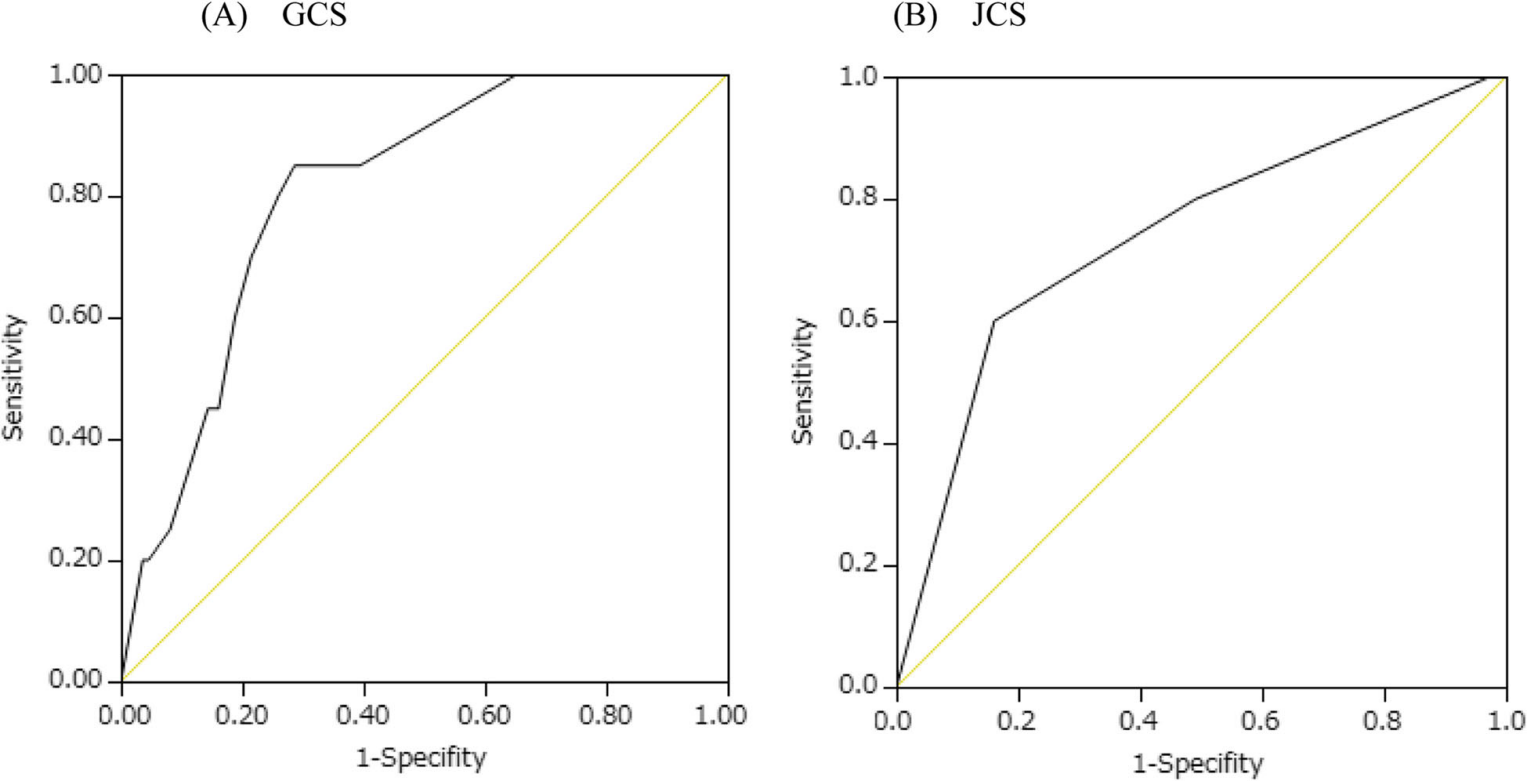
ROC curves of GCS and JCS for prediction of the tracheal intubation. **A** Total GCS (cut-off value: 12, AUC: 0.81, 95% CI: 0.71–0.88, sensitivity: 0.85, specificity: 0.71, *P* < 0.05) and (**B**) JCS (cut-off value: 3, AUC: 0.74, 95% CI: 0.60–0.84, sensitivity: 0.6, specificity: 0.84, *P* < 0.05)

According to ROC curve analysis, GCS and JCS at transport efficiently predicted tracheal intubation in patients with drug overdose (Fig. 1): (A) Total GCS (cut-off value: 12, area under the curve (AUC): 0.81, 95% confidence interval (CI): 0.71–0.88, sensitivity: 0.85, specificity: 0.71, *P* < 0.05), E of GCS (cut-off value: 2, AUC: 0.76, 95% CI: 0.63–0.86, sensitivity: 0.70, specificity: 0.79, *P* < 0.05), V of GCS (cut-off value: 3, AUC: 0.79, 95% CI: 0.68–0.87, sensitivity: 0.75, specificity: 0.74, *P* < 0.05), M of GCS (cut-off value: 5, AUC: 0.76, 95% CI: 0.63–0.85, sensitivity: 0.75, specificity: 0.76, *P* < 0.05) and (B) JCS (cut-off value: 3, AUC: 0.74, 95% CI: 0.60–0.84, sensitivity: 0.6, specificity: 0.84, *P* < 0.05).

### Drugs related to tracheal intubation in patients with drug overdose

The univariate analysis revealed that GC-MS-detected antipsychotics (OR 2.46, 95% CI 1.19–5.20, *P* = 0.01) and anticonvulsants (OR 2.71, 95% CI 1.26–5.98, *P* = 0.01) were significantly correlated with tracheal intubation of patients with drug overdose (Table 2). However, BZs (OR 0.88, 95% CI 0.48–1.46, *P* = 0.64) and antidepressants (OR 1.51, 95% CI 0.35–4.89, *P* = 0.52) were not significant.

**Table 2:** Association between exposure variables and tracheal intubation

	Univariate analysis		Multivariate analysis	
	Crude OR (95%CI)	*P* value	Adjusted OR (95%CI)	*P* value
Antipsychotics	2.46 (1.19–5.20)	0.01*	2.27 (1.07–4.83)	0.03*
Anticonvulsants	2.71 (1.26–5.98)	0.01*	2.50 (1.14–5.64)	0.02*
BZs	0.88 (0.48–1.46)	0.64		
Antidepressants	1.51 (0.35–4.89)	0.52		

* *P* < 0.05

Two variables for multivariate analysis were selected from the results of the univariate analysis and the sample size. According to multivariate logistic analysis, GC-MS-detected antipsychotics (OR 2.27, 95% CI 1.07–4.83, *P* = 0.03) and anticonvulsants (OR 2.50, 95% CI 1.14–5.64, *P* = 0.02) were significantly correlated with tracheal intubation of patients with drug overdose.

### Relationship between tracheal intubation and drug alert blood levels in patients with drug overdose

The alert levels in the blood represent drug concentrations exceeding a certain level, where a significantly increased risk of concentration-related adverse drug reactions can be expected [24–26]. We investigated the relationship between reaching alert blood levels and tracheal intubation (Table 3.). The univariate analysis revealed that GC-MS-detected alert levels of antipsychotics (OR 4.04, 95% CI 0.63–25.9, *P* = 0.14) and anticonvulsants (OR 27.8, 95% CI 2.92–264.1, *P* = 0.004) in blood were significantly correlated with tracheal intubation of patients with drug overdose (Table 3). However, the alert levels of BZs (OR 0.75, 95% CI 0.23–2.43, *P* = 0.63) in blood was not significant.

**Table 3:** Association between exposure variables and tracheal intubation (over the alert levels in blood)

	Univariate analysis		Multivariate analysis	
	Crude OR (95%CI)	*P* value	Adjusted OR (95%CI)	*P* value
Antipsychotics	4.04 (0.63–25.9)	0.14	2.73 (0.32–23.6)	0.36
Anticonvulsants	27.8 (2.92–264.1)	0.004*	24.9 (2.56–241.6)	0.006*
BZs	0.75 (0.23–2.43)	0.63		

* *P* < 0.05

Two variables for multivariate analysis were selected from the results of Table 2 and sample size. According to multivariate logistic analysis, GC-MS-detected alert levels of anticonvulsants (OR 24.9, 95% CI 2.56–241.6, *P* = 0.006) in blood was significantly correlated with tracheal intubation of patients with drug overdose. However, GC-MS-detected alert levels of antipsychotics (OR 2.73, 95% CI 0.32–23.6, *P* = 0.36) in blood was not significant. Antidepressants were not examined because there was no relevant case.

## Discussion

In this study, there are three new findings regarding drugs used by patients with drug overdose due to self-harm requiring tracheal intubation. First, GCS and JCS at transport efficiently predicted tracheal intubation in patients with drug overdose. Second, tracheal intubation and the use of anticonvulsants and anticonvulsants reaching alert blood levels were found to be independently associated with tracheal intubation. The OR was 2.7 times for each type of anticonvulsant taken. Furthermore, it was shown that the OR was 27.8 times higher when anticonvulsants reached alert levels in blood. Third, antipsychotics, which are often used by patients with drug overdose [13, 14, 27, 28], were significantly associated with tracheal intubation, but not when they reached alert blood levels. An overdose of antipsychotics was found to increase the OR for tracheal intubation by 2.5 times. In addition, when multivariate logistic analysis was performed using antipsychotics and anticonvulsants as explanatory variables, the OR for tracheal intubation was 2.3 times higher for antipsychotics and 2.5 times higher for anticonvulsants (for each type). Anticonvulsant drugs included target of therapeutic drug monitoring, such as valproic acid, carbamazepine, and lamotrigine [24–26]. From the results of this study, as many of these drugs reached to an alert blood level due to overdose, it was thought that the adverse effects of respiratory inhibition might occur in patients due to excessive central inhibition. As a result, OR for tracheal intubation was considered to be increased compared with other drugs. Tracheal intubation in patients with drug overdose due to self-harm is determined quickly with limited information. Therefore, the results obtained in this study may be useful information to determine drugs that requires tracheal intubation during triage.

Average body temperature (°C) was almost the same in both groups and was normal. Although there are some missing values, average respiratory rate (breaths/min) was 1–2 times higher than the normal value in all patient groups, but it was close to normal. The average pulse rate (beats/min) was high in patients with drug overdose with tracheal intubation. Antidepressants, antipsychotics, and anticonvulsants may act on the heart and cause tachycardia [29, 30], consistent with the drug results in this study. Therefore, it can be considered that tachycardia was stronger in patients requiring tracheal intubation. In addition, average PO_2_ (mmHg) was higher than the reference value in all patients with drug overdose and was remarkable in patients requiring tracheal intubation. High PO_2_ levels were considered to be the result of tracheal intubation or oxygen administration with an oxygen mask in the time duration between the site of trauma and arrival at the hospital. These values include the results of on-site ambulance crew treatment. Considering these factors, drug overdose often leads to tracheal intubation due to its influence on the consciousness of the patient. In Japan, tracheal intubation is indicated for patients with impaired consciousness with a GCS of ≤8 and a JCS of ≥2, but there is no regulation for patients with drug overdose. In this study, it was clarified that GCS and JCS during transportation appropriately predicted tracheal intubation. However, tracheal intubation for drug overdose may have been required based on the criteria for impaired consciousness, so it is necessary to consider information on the used drug.

Although previous studies reported frequency as a single used drug, the results of this study are similar in that antipsychotics are used more frequently in tracheal intubated patients [13, 14]. On the other hand, the major difference was that the use of narcotics and stimulants as medications to be taken was small in this study. Reports have stated that the use of narcotics and stimulants is relatively low in Japan [31], which is affirmed in this study. In the past, drug overdose in Japan has been reported to be related to barbituric acid-based drugs as drugs that worsen the clinical course, such as long-term stay in the intensive care unit and aspiration pneumonia [31]. Aspiration pneumonia caused by tracheal intubation may extend the length of hospital stay and, as a result, it is likely to use a lot of medical resources [32, 33]. Therefore, anticonvulsants and antipsychotics, which were found to increase the OR for tracheal intubation by overdose in this study, may also have a risk of worsening the clinical course. In addition, anticonvulsants and antipsychotics have the same lethal toxicity and are extremely dangerous in terms of mortality [34]. Anticonvulsants and antipsychotics are prescribed as treatments for bipolar disorder [35, 36]. From the above, it is considered that a relatively serious condition requiring tracheal intubation may occur, especially for patients with bipolar disorder with an overdose of regular medications. Criteria for tracheal intubation when GCS is ≤8 exist for trauma, but there are no criteria for patients with drug overdose [1]. The results of this study may be included in the criteria for considering the need for tracheal intubation based on the drugs used. Furthermore, in clinical practice, it may be possible to prevent drug overdose by paying attention to the appropriate number of drugs in stock and the use of the drug itself according to the number of prescription days, but verification with a larger sample group is required. Toxicity of anticonvulsants and antipsychotics includes central nervous system depression and cardiotoxicity, which are fatal [30, 37–41]. Some reports stated that meropenem is effective as an antidote for an overdose of sodium valproate [42, 43], and an intravenous injection of lipid emulsion is effective in detoxifying cardiotoxicity due to an overdose of quetiapine [44]. However, neither anticonvulsants nor antipsychotics have a specific antidote, and the treatment is symptomatic. For this reason, overdose with anticonvulsants and antipsychotics may require continuous electrocardiogram measurement of the patient as well as respiratory management after tracheal intubation.

As a limitation of this study, only drugs that can be detected by GC-MS are targeted. Two cases could not be detected; the tracheal intubated patients included overdose with lithium carbonate alone (*n* = 1) and overdose with ethylene glycol and psychotropic drugs (*n* = 1) in this study. Ideally, other methods should be combined for the optimal treatment of patients with drug overdose. In addition, in this study, we only investigated each drug category, such as antipsychotics and anticonvulsants. How individual drugs in the drug category are associated with tracheal intubation could not be investigated due to the small number of cases. Although the data of this study is a single result immediately after transport, drug blood levels may be used for on-site triage because they adequately indicate clinical severity [26]. However, it should be noted that differences in the dynamics of individual drugs, such as Tmax and T1/2, are not taken into consideration. For proper treatment, fluctuations in blood levels should be monitored after arrival at the hospital. Since it was a single-institutional result, local community may affect the result. Finally, this study only examined the level of consciousness based on GCS and JCS to what extent each drug influenced patients. Since individual drugs may have different effects on the cranial nerve system and organs, the drugs taken in individual cases should also be considered for appropriate treatment.

## Conclusions

In this study, logistic analysis revealed that the use of anticonvulsants and antipsychotics is significantly associated with an increased OR in the tracheal intubation of patients with drug overdose due to self-harm. The results obtained may be beneficial for the transport triage in the ED.

## Data Availability

The datasets that are either or both used and analyzed during the current study are available from the corresponding author on reasonable request.

## Abbreviations

GCS: Glasgow coma scale
JCS: Japan coma scale
BZs: benzodiazepine receptor agonists
GC-MS: gas chromatography–mass spectrometry
ED: emergency department
ROC: receiver operating characteristic
AUC: Area under the curve
CI: Confidence interval
OR: Odds ratio
